# Metabolic modelling links Warburg effect to collagen formation, angiogenesis and inflammation in the tumoral stroma

**DOI:** 10.1371/journal.pone.0313962

**Published:** 2024-12-03

**Authors:** Maxime Mahout, Laurent Schwartz, Romain Attal, Ashraf Bakkar, Sabine Peres

**Affiliations:** 1 CNRS, Laboratoire Interdisciplinaire des Sciences du Numérique, Universite Paris-Saclay, Orsay, France; 2 Assistance Publique des Hôpitaux de Paris, Paris, France; 3 Cité des Sciences et de l’Industrie, Paris, France; 4 Faculty of Biotechnology, October University for Modern Sciences and Arts, Giza, Egypt; 5 UMR CNRS 5558, Laboratoire de Biométrie et de Biologie Évolutive, Université Claude Bernard Lyon 1, Villeurbanne, France; 6 INRIA Lyon Centre, Villeurbanne, France; Federal University Dutse, NIGERIA

## Abstract

Cancer cells are known to express the Warburg effect—increased glycolysis and formation of lactic acid even in the presence of oxygen—as well as high glutamine uptake. In tumors, cancer cells are surrounded by collagen, immune cells, and neoangiogenesis. Whether collagen formation, neoangiogenesis, and inflammation in cancer are associated with the Warburg effect needs to be established. Metabolic modelling has proven to be a tool of choice to understand biological reality better and make *in silico* predictions. Elementary Flux Modes (EFMs) are essential for conducting an unbiased decomposition of a metabolic model into its minimal functional units. EFMs can be investigated using our tool, *aspefm*, an innovative approach based on logic programming where biological constraints can be incorporated. These constraints allow networks to be characterized regardless of their size. Using a metabolic model of the human cell containing collagen, neoangiogenesis, and inflammation markers, we derived a subset of EFMs of biological relevance to the Warburg effect. Within this model, EFMs analysis provided more adequate results than parsimonious flux balance analysis and flux sampling. Upon further inspection, the EFM with the best linear regression fit to cancer cell lines exometabolomics data was selected. The minimal pathway, presenting the Warburg effect, collagen synthesis, angiogenesis, and release of inflammation markers, showed that collagen production was possible directly *de novo* from glutamine uptake and without extracellular import of glycine and proline, collagen’s main constituents.

## Introduction

In a 1966 lecture to the meeting of Nobel Laureates at Lindau on Lake Constance, Otto Warburg stated, “Cancer, above all other diseases, has countless secondary causes. But, even for cancer, there is only one prime cause. Summarized in a few words, the prime cause of cancer is the replacement of the respiration of oxygen in normal body cells by a fermentation of sugar. Normal body cells meet their energy needs by respiration of oxygen, whereas cancer cells meet their energy needs in great part by fermentation. (…) From the standpoint of the physics and chemistry of life this difference between normal and cancer cells is so great that one can scarcely picture a greater difference. Oxygen gas, the donor of energy in plants and animals is dethroned in the cancer cells and replaced by an energy yielding reaction of the lowest living forms, namely, a fermentation of glucose” [1].

Increased glycolysis such as described by Otto Warburg is seen on PET scan. There is an increased uptake of radiolabelled deoxyglucose by the tumor. This examination is now done on routine basis to assess both how far the cancer has spread and response to treatment such as chemotherapy or immunotherapy [2].

There is still debate on the definition of cancer. Sonnenschein and Soto proposed that cancer might be the consequence of tissue disorganization [3]. These two authors suggested that carcinogenesis is a problem of tissue architecture, comparable to organogenesis during early development, and that proliferation is the default state of all cells. The Warburg effect was first described on isolated cells as an “aerobic glycolysis” fermentation process with increased secretion of lactic acid. Whether this glycolysis is responsible of changes at the tissue level still needs to be elucidated.

Macrophages and myeloid cells are key players in the activation of the tumor-associated stroma. Macrophages, once established in the tumor stroma, play critical roles in stimulating angiogenesis [4]. Extracellular secretion of cytokines, such as IL1*β* or TNF*α*, and lymphokines is noticeable in the tumor stroma, as well as the presence of growth factor VEGF-A, which is linked to tumor blood vessel formation [5]. On the other hand, collagen is the most abundant protein in the extracellular matrix (ECM) and is essential for tissue architecture and function [6]. Changes in collagen synthesis, deposition, and organization have been linked to tumor progression and metastasis in cancer. Collagens and many other extracellular matrix molecules are primarily produced by fibroblasts in tumors, and recent evidence suggests that tumor-derived collagens play a role in tumor progression and metastasis [7].

In addition to glucose, glutamine has been found to be another key nutrient for cancer cells. Glutamine may participate in the formation of collagen via different pathways [8–10]. It is not known however if this is related to the Warburg effect [11]. As well, it has been shown that lactic acid may act as an activator of one of the collagen-forming enzymes [12]. Hence, relationships between Warburg effect and collagen need to be clarified. Whether inflammation and angiogenesis are involved, since they are key players in the tumor microenvironment, also needs to be investigated.

To gain deeper insights into the complex pathways involved in cancer metabolism, appropriate modelling methods must be used. In particular, metabolic modelling techniques are used to represent cellular reactions within network structures for predicting outcomes through computer-based analysis. Metabolic pathways of medical relevance such as the Warburg effect can be observed on these network models [13].

Constraint-based modelling is a subdomain of metabolic modelling where the steady-state assumption applies: variations over time of metabolite concentrations are not modelled. The modeller is interested by metabolite quantities going into exchange reactions—metabolite uptake or secretion—and in each reaction internal to the cell [14].

Flux Balance Analysis (FBA) is an optimization procedure allowing to compute metabolite flux values for each reaction of the metabolic network model, in maximizing or minimizing an objective function [15]. Usually the function to maximize is flux through a biomass reaction simulating cellular growth. In the context of human cancer cells, the validity of such an optimization procedure might be discussed [16].

Standard FBA is not sufficient to be representative of cells minimizing their limited number of resources. Parsimonious FBA (pFBA) proposes to minimize the number of reactions found within a FBA solution [17]. However, as optimization methods, pFBA and FBA do not account for all possible alternate pathways.

An alternative method proposed—outside of an optimization scope—would be to retrieve all minimal possible flux pathways of the network, in an enumeration procedure. This idea entails the calculation of Elementary Flux Modes (EFMs), a formalism that characterizes the all minimal sets of enzymes from a metabolic model operating at steady-state with all irreversible reactions proceeding in the appropriate direction [18, 19]. The method differs from flux sampling, which consists in sampling linear program solutions [20]: EFMs are extremal rays of the linear programming space.

After enumeration, EFMs are typically ranked and sorted, the less biologically significant ones being excluded [21, 22]. EFMs analysis allows for a complete description of the network and its possible flux distributions if all EFMs are enumerated, but unfortunately it is hardly possible computationally to get all solutions as the size of the metabolic network grows [23, 24].

As a result, tools are being developed to enumerate only a handful of EFMs, a subset of solutions of biological relevance [25, 26]. Recently, we have developed a powerful hybrid tool called *aspefm* [26] that can efficiently compute subsets of Elementary Flux Modes (EFMs) on metabolic networks while considering various biological constraints such as thermodynamic equilibrium, transcriptional regulation, and biomass operating costs.

In this analysis, we harness the power of the *aspefm* method to compute EFMs in accordance with exometabolomics data of 60 cancer cell lines on a medium-scale metabolic model. We design constraints to select pathways linking the Warburg effect to tumoral growth, collagen production, neoangiogenesis and inflammation response. We illustrate that EFMs analysis yields a diversity of pathways among which we can select the most-fitting ones to clinical data. Results of linear regressions are compared with parsimonious FBA and flux sampling, showing that EFMs analysis yield the best-scoring pathways. Leading to insight into collagen production from glutamine, the retrieved pathways enable us to propose a possible explanatory scenario for the production of the tumoral stroma.

## Results

### Model description and methodology

In order to show our prospect that production of lactate from glucose, collagen from glutamine and release of cytokines are linked together with tumoral growth in cancer cells, we computed Elementary Flux Modes (EFMs) on a modified version of a core metabolic model of central human metabolism: the C2M2NF model by Mazat and Ransac [27, 28].

C2M2NF, Central Carbon Metabolic Model with added Nitrogen and Folate, is a reduced metabolic model of central carbon metabolism comprising about a hundred reactions and metabolites total. The model possesses three compartments, external, cytoplasmic and mitochondrial. It includes an oxidative phosphorylation (OxPhos) reaction system, as well as mitochondrial transporters with pseudo-metabolites (DPH and DPSI) representing the proton gradient through the mitochondrial membrane. Another pseudo-metabolite (PMFm) derived from this gradient is used to represent the mitochondrial protomotive force. Finally, the model possesses a biomass reaction, modelling tumoral growth.

In this work, we added amino acid transporter reactions for the remaining amino acids not considered in C2M2NF, reactions related to amino acid degradation, synthesis of collagen and inflammatory response markers, and thus obtain our own version of the model, which we could call C2M2NFS, for Central Carbon Metabolic Model with added Nitrogen, Folate, and Stroma formation. Stroma formation is itself characterized by collagen synthesis, inflammatory response markers IL1*β* or TNF*α*, and growth factor VEGF-A, linked to neoangiogenesis.

We provided the C2M2NFS model in SBML format in S1 File. An important point of note is that, as in C2M2NF, for simplicity, metabolism of the following amino acids: (Asp and Asn), (Thr, Iso, Val), (Tyr, Phe, Leu, Lys, Trp) are conflated together. In particular, the latter two groups are combined into single metabolites: TIV and YFLKW, and their uptake (TIVUP, YFLKUP) or catabolism (TIVDG, YFLKWDG) are defined by single group reactions [28]. This is beneficial for EFMs analysis as it allows for working with a smaller-scale model.

Given these informations, in a similar vein to the analysis conducted in Mazat [28], we retrieved exometabolomic data from Jain et al on the NCI-60 cell lines [29], including an estimation of exchange fluxes for a total of 60 cancer cell lines in fmol / cell / h. Considering the standard deviation and mean exchange fluxes of the cellular lines, we separated the global experimental observations: uptake, secretion, or both, into two categories of constraints for our computation: hard constraints, what we force as an input constraint for our computation, and desired observations, inputs we expect to observe in the minimal pathways, but do not force. The resulting constraint data is reported in Table 1.

**Table 1:** Mean +/- SD exchange fluxes intervals from NCI-60 exometabolomics data. Includes glucose, lactate, XTP, pyruvate, formate, and amino acids, from which we derive constraints for our model. Minus (-) symbolizes uptake while plus (+) symbolizes secretion. Experimentally induced constraints are separated into two kinds: hard constraints (red bold font) and desired observations (normal font), indicating different types of logical constraints.

Metabolite	Glucose	Lactate	Glutamine	Glutamate	Serine	Glycine	Alanine	Proline	Asp Asn	Arginine
Mean +/- SD cancer cell exchange flux interval	-326.87 +/- 196.12	442.20 +/- 289.40	-82.48 +/- 56.20	13.54 +/- 16.99	-11.57 +/- 7.05	0.96 +/- 2.97	15.89 +/- 13.34	1.21 +/- 1.49	-3.33 +/- 3.39	-4.90 +/- 4.44
Corresponding constraint	–	+	–	+	–	+/-	+	+/-	–	–
Metabolite	TIV (Thr, Ile, Val)	YFLKW (Tyr, Phe, Leu, Lys, Trp)		Nucleotides (XTP)		Pyruvate	Formate	Histidine	Cysteine	Methionine
Mean +/- SD cancer cell exchange flux interval	-14.90 +/- 7.99	-19.80 +/- 10.57		0.10 +/- 0.22		Uncalibrated data	Data missing	Data missing	0.05 +/- 0.08	-2.11 +/- 1.23
Corresponding constraint	–	–		+/-		+	+/-	–	+/-	+/-

Encoding ‘hard constraints’ and ‘desired observations’ as logical constraints for the computation of EFMs, we were able get subsets of EFMs presenting the desired phenotypes. Warburg effect and glutamine uptake were included as ‘hard constraints’, while the rest of experimental observations were included as ‘desired observations’.

As well, only EFMs presenting flux in all of three metabolic functions: cell proliferation, stroma response and collagen formation, were sought for. And in particular, we required through linear constraints that more flux is going into collagen synthesis and factors recruitment than biomass. Finally, a size limit constraint was added, helping to limit flux utilization. This allows for enumerating a smaller subset of minimal pathways observed with EFMs.

Note that the ‘hard constraints’ on Warburg effect and glutamine uptake coupled with the linear constraints on biomass growth, inflammation and collagen production makes it so that all EFMs obtained contain Warburg effect and tumoral growth—as well as glutamine uptake, inflammation and collagen production. There are still uncertainties on how Warburg effect and tumoral growth are linked [30, 31].

### Minimal pathways linking Warburg effect to collagen production, inflammation and angiogenesis markers production show high variability in usage of the tetrahydrofolate cycle

**Fig 1:**
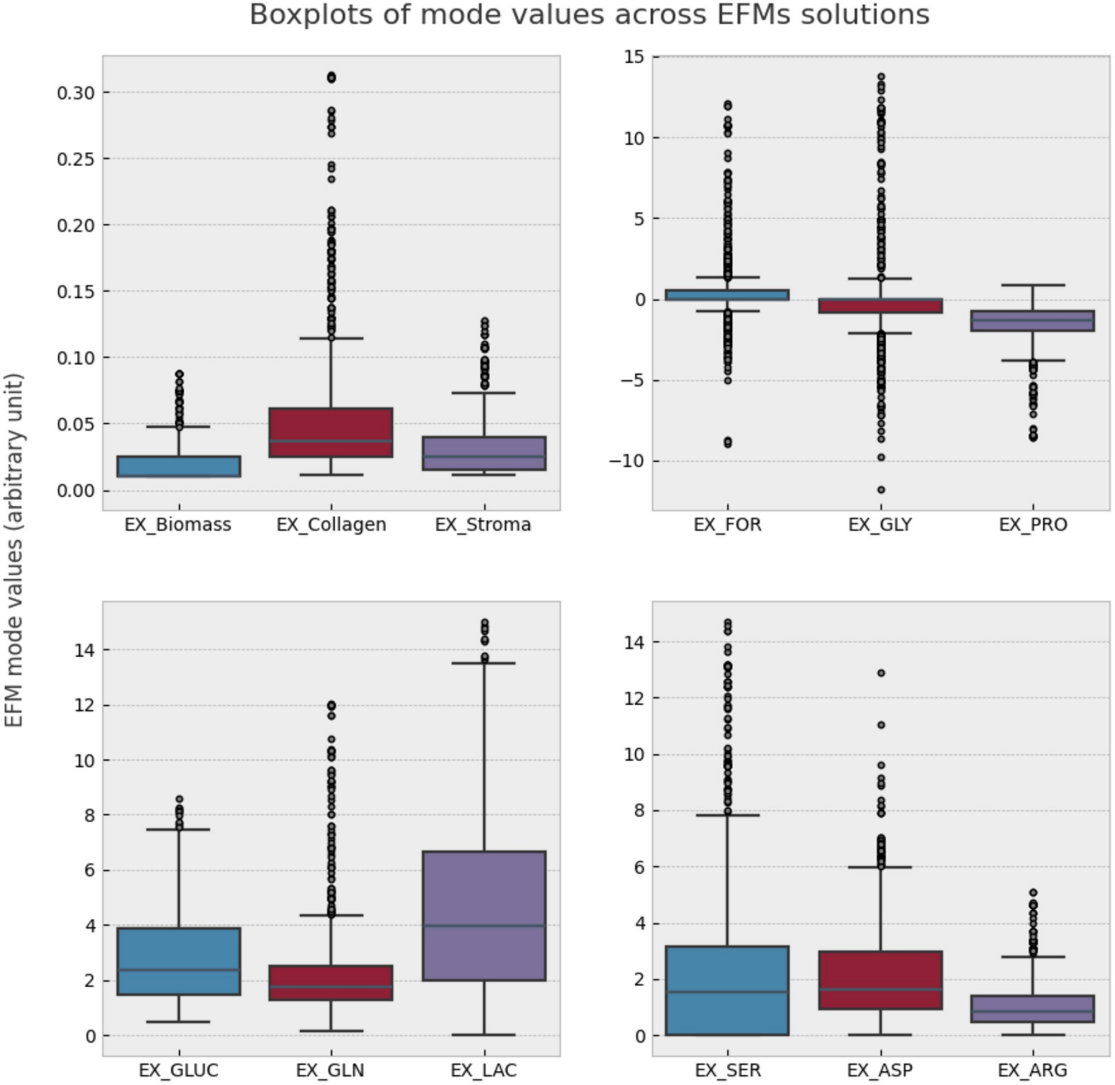
Statistics of our principal reactions of interest among all 747 EFMs. EX_Biomass: tumoral growth, EX_Collagen: collagen production, EX_Stroma: recruitment of inflammation markers IL1*β* or TNF*α*, and growth factor VEGF-A, EX_GLUC: glucose consumption, EX_GLN: glutamine consumption, EX_LAC: lactate production, EX_FOR: formate consumption or production, EX_GLY: glycine consumption or production, EX_PRO: glycine consumption or production, EX_SER: serine consumption, EX_ASP: aspartate and asparagine consumption, EX_ARG: arginine consumption. In the case of EX_FOR, EX_GLY, EX_PRO, negative values represent consumption, and positive values represent production.

After running enumeration of EFMs with *aspefm* for 3.5 days, a sample of 747 unique minimal pathways were obtained. These metabolic pathways spanned a large diversity of possible inputs and outputs, including the varying flux yield values of metabolic reactions. The statistics of yield values of the principal reactions of interest were plotted in Fig 1. A complete view of the statistics of exchange yields in our EFMs can be found in S1 Table.

As demanded by our constraints, all obtained EFMs showed Warburg effect, glutamine consumption, tumoral growth, production of collagen, either inflammation markers IL1*β* or TNF*α*, representing cell inflammation, and growth factor VEGF-A, representing neoangiogenesis. We provided the obtained EFMs in S2 File.

For ‘desired observations’, the EFMs either respected observations from Table 1, or did not input any flux into the exchange reactions. Despite not being ‘hard constraints’, some amino acids clusters were observed to be consumed in all minimal pathways for tumoral growth and stroma production: cysteine, histidine, TIV and YFLKW. These particular cases were confirmed to be essential reactions with our constraints using FBA.

Conversely, consumed-only amino acids arginine, serine, aspartate and asparagine, were more often than not absent from consumption by EFMs. Similarly, secreted-only amino acids including glutamate and alanine often did not appear to be produced. Among the most common motifs that compose variability in reactions in EFMs, we noticed four vastly fluctuating exchange reactions (S1 Table) that appear related to usage of the tetrahydrofolate (THF) cycle: formate (SD: 2.40), glycine (SD: 3.36), methionine (SD: 0.60), and serine (SD: 3.30).

### EFMs analysis displays stronger results in linear regression to mean flux data than parsimonious FBA and flux sampling

**Fig 2:**
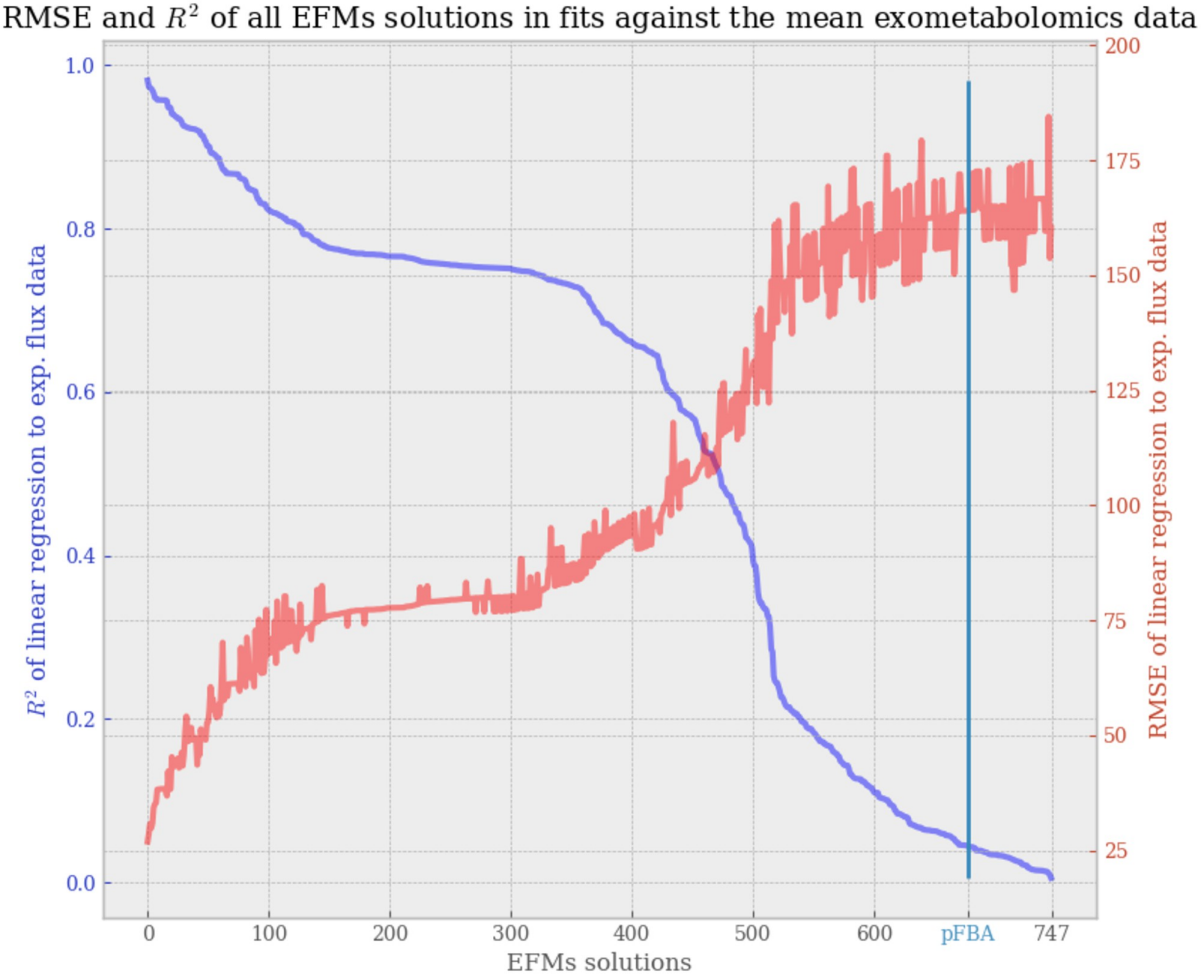
RMSE and R^2^ scores of linear regressions to mean NCI-60 flux data, performed on all 747 EFMs solutions. Indicated by a vertical cyan line, the R^2^ score of the pFBA solution was reported on this graph for comparison with the R^2^ of the EFMs solutions.

For each elementary mode obtained with our analysis, we performed a linear regression of their non-null uptake mode values to the corresponding mean uptake fluxes of the NCI-60 cancer cell lines, as were reported in Table 1. A large diversity of regression scores for EFMs analysis is observed, ranging from a R^2^ of 0.005 to a R^2^ of 0.98, and from a RMSE of 26.9 to a RMSE of 184.5. The analysis of the 747 EFMs, reordered by linear regression score, is summarized in Fig 2.

As discussed previously, the validity of using Flux Balance Analysis for modelling healthy and tumoral cells can be controversed [16], and in our case we aim to model not just one metabolic function of the cell, growth, but three simultaneously: growth, production of collagen, and production of stroma markers. For comparison to EFMs analysis, a solution to Parsimonious Flux Balance Analysis (pFBA) with the same constraints was computed. We added a marker in Fig 2 for the score obtained by pFBA in the same linear regression against the mean exometabolomics data.

The pFBA solution is visualized with EscherPy in S2 Fig. In the pFBA solution, a clear bias is seen towards the objective function, which was set to sum of production fluxes BM, COLLAG and STRO. As an example, the flux of reaction CBS is saturated to 15.0 which is the upper bound set to every flux, and three more amino acids were found imported than for the optimal EFM solution. Both inflammation markers IL1*β* and TNF*α* are produced, while only one of those could have been necessary at a given time. Warburg effect’s lactate over glucose ratio is of -0.08, which does not match the ratios of the best scoring EFMs and of the mean exometabolomics data, of around -1.35, possibly explaining the pFBA solution’s regression score around the worst 10% of EFMs. These characteristics of the pFBA solution show that relying on an objective function is not always the most appropriate choice. Meanwhile, EFMs analysis performs no maximization and returns a diverse panel of minimal pathways that the cell can alternate between at each given time.

**Fig 3:**
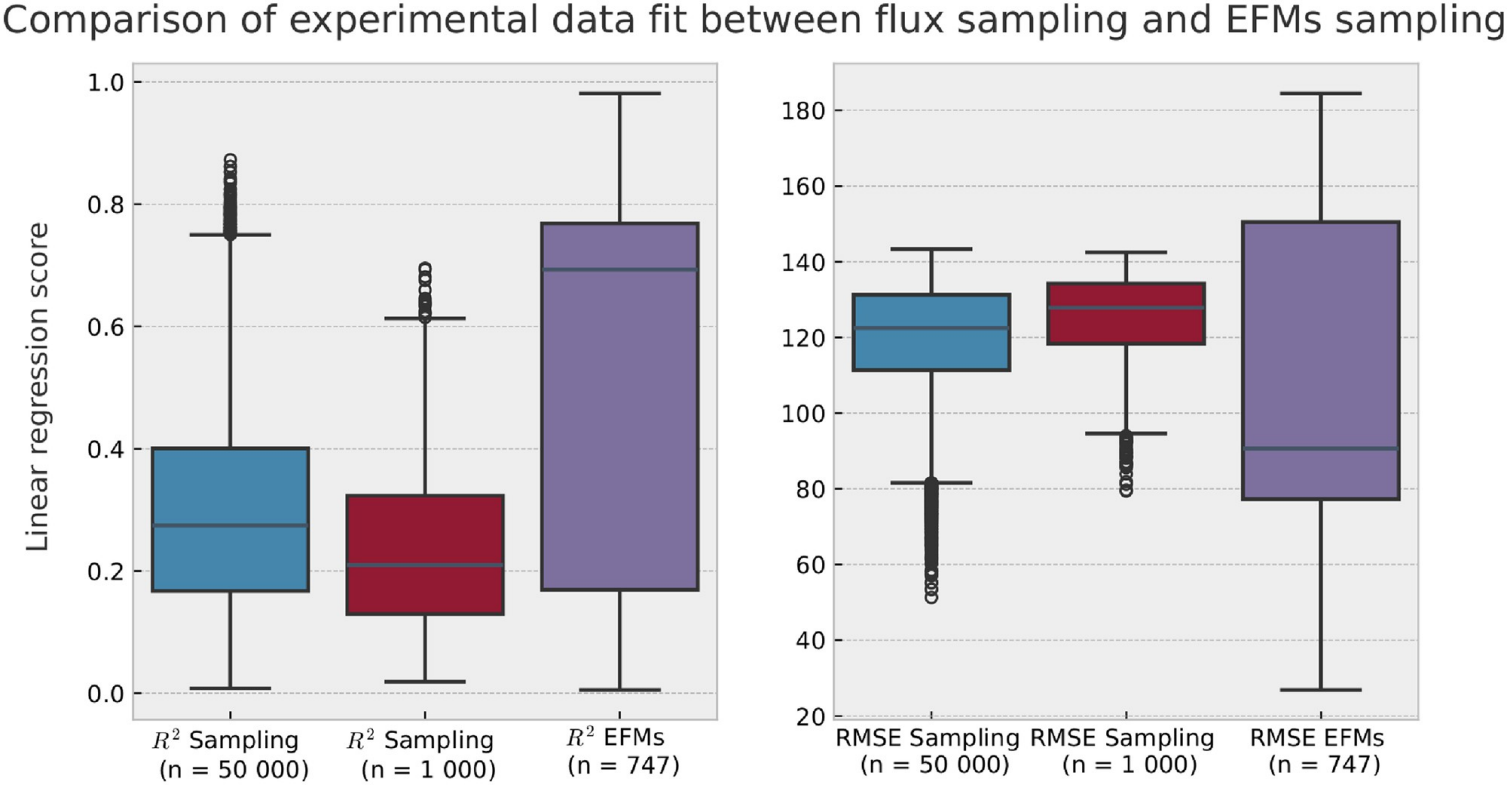
Comparison of exometabolomics data fit between flux sampling and EFMs sampling. Boxplots of regression score values—RMSE and R^2^—for fitting to mean NCI-60 flux data, for the flux sampling of 1 000 or 50 000 solutions obtained with OptGPSampler, and using the 747 EFMs computed with *aspefm* in this study.

A better suited comparison to EFMs analysis than pFBA could be flux sampling [20]. Therefore, we also performed flux sampling on our model and linear regression of non-null exchange fluxes of all flux sampling solutions towards mean NCI-60 dataset exometabolomics data. We chose flux samples of size *n* = 1000 and *n* = 50000. We present the results of the regressions in Fig 3. Flux sampling achieves a maximum R^2^ to mean exometabolomics data of 0.70 with a sample of 1 000 solutions, and a maximum R^2^ of 0.87 with a sample of 50 000 solutions. While, with a sample of 747 EFMs, we are able to reach a maximum R^2^ of 0.98. We also observe that EFM analysis achieves both the highest and lowest scores of RMSE, suggesting a seemingly wider variety of solutions calculated, although not all EFMs respecting the constraints were computed. Unlike sampled fluxes, EFMs are indeed, by nature, extremal solutions.

### Optimal EFM fit to mean exometabolomics data exhibits *de novo* synthesis of collagen from glutamine, without uptake of proline or glycine

**Fig 4:**
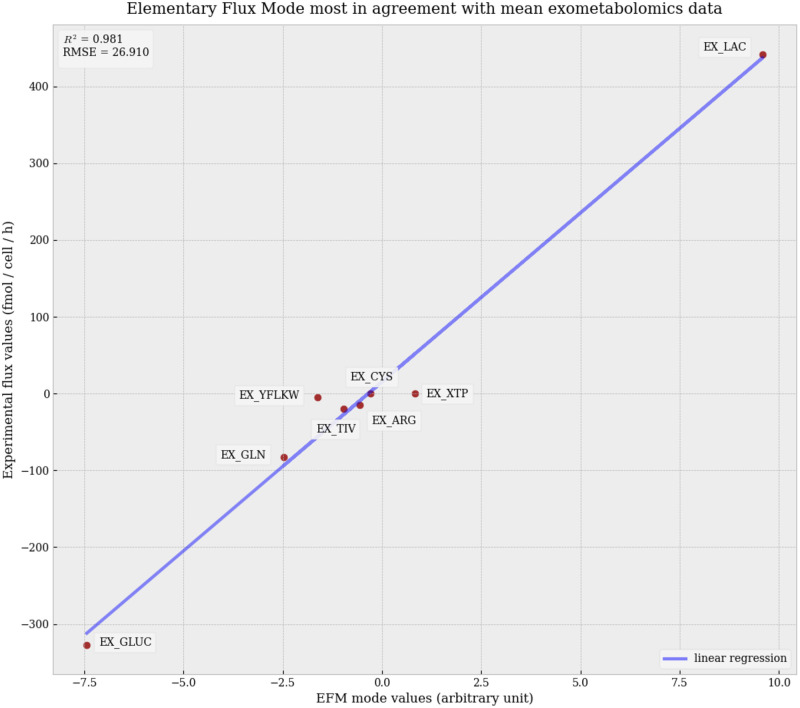
Linear regression of the Elementary Flux Mode with the best fit to mean NCI-60 cancer lines flux data. Represented reactions correspond to exchange reactions with non-null EFM values.

**Fig 5:**
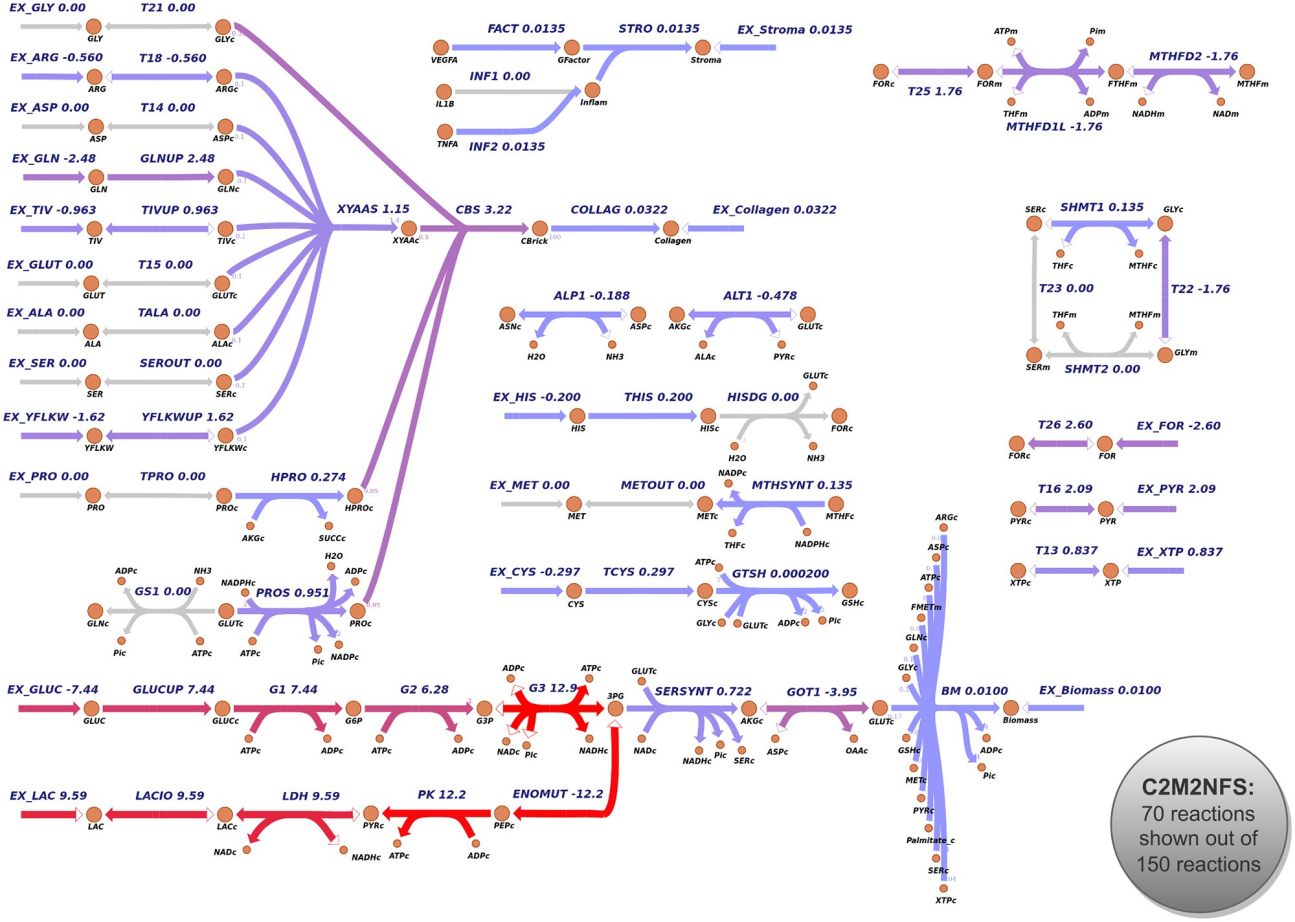
EFM with the best fit to mean exchange fluxes from NCI-60 exometabolomics data. 70 reactions of most interest of C2M2NFS are shown, including most cytosolic transporters and some mitochondrial transporters but not mitochondrial TCA Cycle. Visualization of the reactions is done through the EscherPy Python package.

In order to derive a single pathway of interest, we should select the EFMs that best fits the mean exometabolomics data. We provide in Fig 4 the linear regression analysis of the EFM that best fits the mean exometabolomics data, with scores of R^2^ of 0.98 and RMSE of 26.9. Principal reactions of the EFM were also represented in Fig 5 with the EscherPy software [32]. Notably, this EFM is numbered 412 among our 747 EFMs.

The optimal metabolic pathway is characterized by the secretion of TNF*α* as the inflammatory response marker, VEGF-A as a representant of neoangiogenesis, consumption of the following amino acids: glutamine, cysteine, histidine, arginine, TIV, and YFLKW; collagen production and Warburg effect. Nucleotide synthesis is performed above biomass requirements which results in nucleotide secretion. The tetrahydrofolate cycle is used through cytosolic reaction SHMT1, and mitochondrial reactions MTHFD1L, MTHFD2.

No external consumption of glycine or proline is observed, indicating that in the case of this elementary pathway, amino acids glycine and proline, going into collagen synthesis and representing about 50% of the collagen content, are synthesized *de novo*, purely through other metabolic reactions and catabolism of other amino acids.

In particular, fluxes of notice include high glutamine consumption (-2.48), which is converted into 2.01 units of glutamate through nucleotide synthesis by the NUC reaction (0.87). Glutamate is also obtained from *α*-ketoglutarate (*α*KG) using the GOT1 reaction (-3.95). From these 5.96 units of glutamate, 3.25 units go into mitochondria and get converted into *α*KG, from which 0.24 units are transformed to citrate through reverse tricarboxylic acid cycle usage, then to oxaloacetate and acetyl-CoA, contributing to biomass lipids production. Among the remaining cytosolic glutamate units, 0.72 units are converted into serine by SERSYNT, 0.95 units are used for proline production, 0.48 units are used for alanine synthesis, 0.43 units are used for VEGF-A and TNF*α* production, 0.11 for collagen formation. And from the produced serine units, 0.13 units are converted into glycine through the use of SMHT1.

Thus, glutamate can be converted into proline by reaction PROS, hydroxyproline through reaction HPRO, and glycine through reaction SERSYNT and then use of the THF cycle with reaction SHMT1 to convert serine into glycine, making up for the three principal collagen constituents. We believe that the best EFM fit not importing both of those collagen constituents but making use of glutamine—converted to glutamate and serine—to produce proline and glycine *de novo* is a result of major importance shown by our model and our methodology.

### Best scoring EFM is shared among the majority of cell lines, and is aspecific to particular cancer types

To assess the validity of our optimal EFM for the cancer cell lines, we pushed the analysis further, calculating the linear regressions of non-zero uptake reaction values of EFMs to the singular exometabolomics data of each cell line, instead of to the averaged data as done previously. This will also allow us to investigate whether certain cancer types are more prone to using this EFM or another.

**Fig 6:**
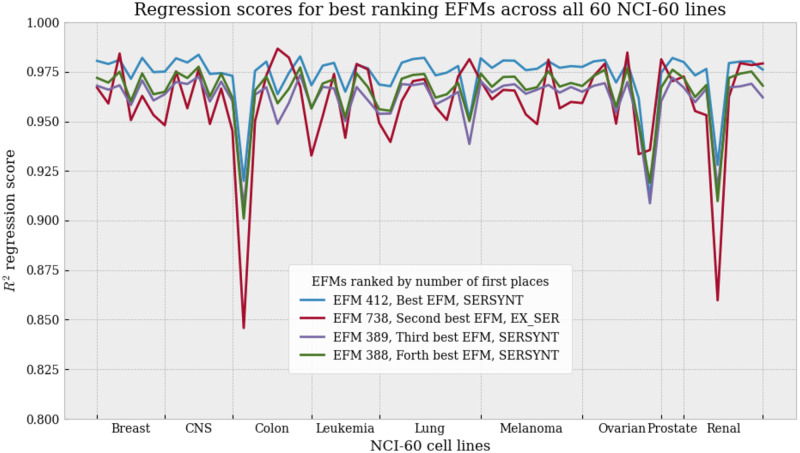
Linear regression scores for best ranking EFMs across all NCI-60 lines. Plot of *R*^2^ linear regression scores for 4 top ranking EFMs, over all sixty cell lines, sorted by cancer cell types. CNS: Central Nervous System. SERSYNT: usage of intracellular serine synthesis reaction. EX_SER: extracellular serine import. Here the two reactions are mutually exclusive.

As it turns out, our best ranked EFM against the mean exometabolomics data, EFM 412, also ranks best overall against all sixty cell lines, with most amount of first places. We represent the linear regression scores for the 4 best-ranking EFMs against all 60 cell lines in Fig 6. Of the linear regressions, EFM 412 arrives in first place for 42 out of 60 cell lines, EFM 738 arrives in first place for 14 solutions, and EFMs 388, 389 and 393 (not pictured, $\bar{R^{2}}=0.882$) share the remaining 4 first places. Interestingly, if instead of counting the number of first places, average regression score across all sixty lines is compared, then EFM 412 is still first with $\bar{R^{2}}=0.973$, but EFM 388 is second with $\bar{R^{2}}=0.965$, EFM 389 is forth, with $\bar{R^{2}}=0.961$, and EFM 738 is fifth with $\bar{R^{2}}=0.960$.

Most importantly, EFM 738 is the first suboptimal EFM to replace the intracellular use of SERSYNT by extracellular import of serine with EX_SER. On average, the first 4 EFMs with intracellular serine production are better suited for the variety of cell lines proposed, but for 14 particular cell lines, EFM 738 surprisingly scores above EFM 412.

We were not able to determine in which ways the flux values differed between the cell lines on which EFM 412 had the highest regression score and the cell lines on which EFM 738 had the best scores. However, we suggest that slight differences in flux yields, including Warburg effect yield, glutamine over glucose ratio, and in nucleotide and amino acids consumption, including serine, glycine and proline, might be decisive for a selected cell line to switch between the use of SERSYNT and the use of EX_SER.

In EFM 738, since SERSYNT is replaced by EX_SER, collagen constituant glycine is synthesized from extracellularly imported serine. In even lower-scoring EFMs, in addition to using EX_SER, the cell utilizes exchange reactions EX_GLY and EX_PRO to gather the collagen constituants, although import rates observed on the model do not match with well experimental data values.

In conclusion, EFM 412, where serine, glycine, and proline are synthesized *de novo*, is the best scoring EFM of our model’s analysis among all sixty cell lines. Fig 6 suggests that the EFM is aspecific to any particular cancer type (*e.g*. Renal, Prostate, etc.). We further inspected specificity to all cancer types by computing again which EFM obtains the best linear regression scores, this time against averaged data for each cancer type. Expectedly, EFM 412 scored as first in terms of *R*^2^, regardless of the cancer type. Regression scores for EFM 412 against cell types are given in S2 Table.

### A closer look at tumoral stroma production in the light of Warburg effect

In this work, we selected a subset of EFMs, which displays Warburg effect, and rates of collagen production, inflammatory markers and angiogenesis markers synthesis constrained to be above biomass production. We proposed a novel methodology in metabolic modelling, different from the usual hypothesis that reaction fluxes in the cell only contribute to optimizing its growth. We specifically chose not to integrate the functions of synthesizing collagen and recruiting VEGF-A, IL1*β*, and TNF*α* into the biomass, as we believe these are separate cell functions, and that for a tumoral cell getting rid of its excess nutrients, namely glucose and glutamine, through tumoral stroma formation, is a priority over proliferation.

Our methodology could highlight, in accordance with experimental data, that in a tumoral cell undergoing Warburg effect, several interchangeable pathways using different variable combinations of amino acids were available for the synthesis of our four proteins of interest—collagen, VEGF-A, IL1*β*, and TNF*α*. Note that Jain and coauthors showed that glycine, serine and the THF cycle held a pivotal role in cancer cell metabolism [29]. We computed EFMs according to their exometabolomics data, and our model’s solutions also corroborated these hypotheses.

Additionally, by taking the EFM most in accordance with physiological data we found that glycine, hydroxyproline and proline, the three major components of collagen, could be synthesized endogeneously solely from glutamine, which is converted to glutamate in our model by using the nucleotide synthesis reaction. It is well-known that glutamine-derived glutamate can be converted into proline through 1-Pyrroline-5-carboxylic acid, and then to hydroxyproline [9, 10]. Then, the use of serine synthesis from glutamate and THF cycle usage would allow the cell to obtain its glycine requirement to synthesize collagen, presenting a phenotype typical of the one observed in the stroma around cancer cells. No extracellular proline, glycine or serine import would in fact be needed.

**Fig 7:**
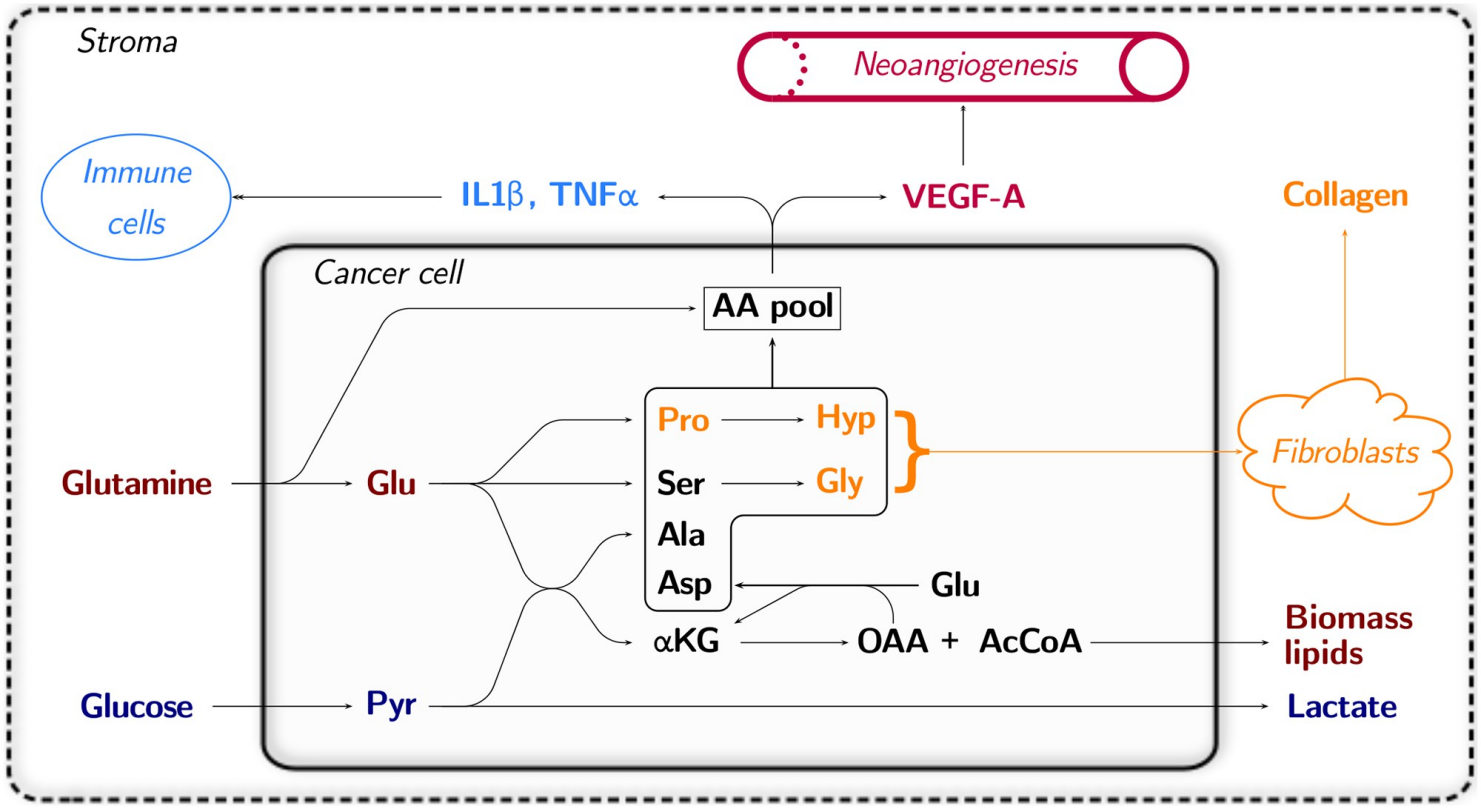
Explicative model of tumoral stroma production in light of amino acid metabolism and the Warburg effect. Two parallel pathways are observed, glycolysis and glutaminolysis. Abbreviations are amino acids three letter codes, Hyp: hydroxyproline, AA: amino acids, *α*KG: *α*-ketoglutarate, Pyr: pyruvate, OAA: oxaloacetate, AcCoa: acetyl-CoA.

In light of these findings, we devised a graphical model of how tumoral stroma might be conceived, presented in Fig 7. The graphical model includes the main findings observed in our optimal pathway represented in Fig 5, namely: glucose is fermented into lactate through glycolysis, and glutamine is converted into glutamate, which is transformed into the main amino acids for collagen production, and into acetyl-CoA lipid bricks helping tumoral growth. Finally, the amino acid pool formed through amino acid biosynthesis and amino acid uptake is used to synthesize inflammation and neoangiogenesis markers, helping to recruit the cells composing the tumoral stroma.

It is interesting to note that the two renowned cancer hallmarks, aerobic glycolysis and glutaminolysis, undergo parallel pathways, glycolysis leading to lactate acidifying the tumoral stroma medium and glutaminolysis leading to increased production of biomass lipids and collagen. Finally, we would like to address an issue that could be raised seeing that standard tumoral cells are not specialized in producing collagen like fibroblasts. Whether or not collagen is produced by the main tumoral cell or by cancer-associated fibroblasts [33], and whether or not fibroblasts themselves undergo Warburg effect as well, as has been hypothesized [34, 35], is not an issue to our metabolic modelling analysis. Indeed, our model could alternately represent a cancer-associated fibroblast producing collagen, or a neighbouring tumor cell which secretes glycine, proline, and hydroxyproline in excess into the fibroblast medium.

## Discussion

Over the years, interest in the constraint-based modelling community has mostly been oriented more towards Flux Balance Analysis-related methods than Elementary Flux Modes, as the latter are effectively more time-consuming and expensive to compute. However, Flux Balance Analysis has the drawback of partaking in maximization of an objective function, which is an assumption that cannot necessarily correlate with biological observations. The solution obtained from Parsimonious Flux Balance Analysis (pFBA), a bilevel optimization problem with minimization of the sum of reactions on top of maximization of the objective function, was unable to fit to the experimental data (R^2^ = 0.044), scoring as low as the ten percent worst EFMs (Fig 2, S1 Fig). Thus, in the case of this application to cancer, we argue that the maximization approach, optimizing the sum of flux going into tumoral growth, collagen formation and inflammation markers production, was not an adequate answer to our problem. As well, one might suggest the use of flux sampling rather than EFMs, with methods such as OptGpSampler [20]. However, perhaps due to the different nature of solutions sampled, we found that flux sampling also did not answer to our problem quite as well as EFMs: achieving a maximum R^2^ score of linear regression to mean exometabolomics data of 0.87 with a sample of 50 000 solutions (Fig 3), while EFMs analysis reached a maximum R^2^ of 0.98 with a sample of 747 EFMs. Therefore, we argue there is a clear need for Elementary Flux Modes analysis, possibly constrained rather than exhaustive, to sample biologically relevant subsets of EFMs.

Although our C2M2NFS metabolic model of 150 reactions would be considered of a relatively small scale by today’s standards, enumeration of EFMs using EFMTool could not finish or yield any results at all [23]. This due to the way its implemented algorithm, Double Description, works [36]. Previous attempts on networks of around this size had to split the model in multiple smaller networks to complete enumeration [37], or resorted to MILP, which may be restricted by minimizing solution size [25]. Another kind of method emerged for networks over this size using Lexicographic Reverse Search [38]. On the other hand, *aspefm* can yield results on metabolic networks of this size and over, up to thousands, very easily, and it is able to handle biological constraints, which is ultimately the goal in EFM analysis. The tool can yield any new EFM in reasonable time—however, downsides appear from choosing constraints that are too difficult to filter out, which unfortunately includes most linear ones [39, 40]. When that is the case, *aspefm* spends most of its time filtering out solutions that are not EFMs instead of finding EFMs, and its enumeration must thus be eventually stopped with a time limit—in our case 3.5 days. These are points of major improvement for our tool in the future.

The debate on whether cancer is a genetic disease, a pathological ecosystem [41] or merely that cancer cells are unable to oxidize glucose properly and rely on fermentation is still not settled. Warburg postulated that cancer relies heavily on glycolysis and that pyruvate is preferentially converted to lactate instead of continuing to Krebs cycle to be converted to ATP by the oxidation phosphorylation where oxygen is needed [42]. This phenomenon—“aerobic glycolysis”—is now recognized as a hallmark of cancer [4, 43]. On the other hand, it has been shown that the stroma plays a key role in tumor development and progression. The stroma is marked by the appearance of cytokines such as IL1*β* and tumor necrose factor TNF*α*, and growth factor VEGF, causing recruitment of new immune cells and blood vessels [43]. As well, glutamine metabolism is accelerated in cancer cells as opposed to healthy, non-proliferating cells, consequently the demand is larger to meet the energy and biomass production needs [44, 45]. Combining calorie-restricted ketogenic diet with glutamine targeting in late-stage experimental glioblastoma has shown clear therapeutic benefits [46]. The triple helix shape of collagen is made up of three polypeptide chains that are coiled around one another. Glycine, proline, and hydroxyproline residues are prevalent in these chains [47]. The production of hydroxyproline, which is necessary for the stability of the triple helix collagen structure, is facilitated by glutamine [8, 48, 49]. Interestingly, our model finds that endogenous collagen production is possible from glutamine only, with no glycine or proline uptake requirement. This result strongly suggests a possible key role of glutamine in the formation of collagen in cancer.

Mazat and Ransac’s model, C2M2NF, which we extended for this study, proves to be a great tool for exploring ‘aerobic glycolysis’ and glutamine metabolism [27]. Similarly to a study by Mazat [28], we retrieved the exometabolomics cancer cells dataset by Jain and collaborators. In their dataset, Jain and collaborators categorize uptake and secretion fluxes of sixty cell lines by their origin tissue [29]. To determine the EFM best in agreement with experimental data, we took the EFM with best linear regression fit to mean flux values of all cell lines regardless of their origin tissue. To further the analysis, we took our optimal EFM, and tested linear regressions against only specific types of cell lines, and finally against all cell lines. We found that our EFM showed no specificity to any of the tumor cell lines, achieving first ranking scores in almost all cases (Fig 6, S2 Table). Alternatively, the issue of tissue specificity, which is of great interest for our study, could be achieved by using larger-scale organ-specific metabolic models [50].

While this EFMs analysis is modelling at a medium-scale level, it should be kept in mind that selected constraints apply. Selected biological constraints are of major importance for us to keep the number of solutions to manually analyze low. And *aspefm*’s specialty is its ability to account for many constraints [26]. Yet the analysis being stopped after a time limit, and constrained rather than exhaustive, means that some elementary metabolic pathways also descriptive of biological processes of interest might have been filtered out by our methodology. It should also be noted that the steady-state assumption for intracellular metabolites is a strong hypothesis, ignoring all internal thermodynamic and time-dependant processes at play. Finally, other modellers might consider a smaller-scale level analysis such as the one presented in Braakman and Smith [51] appropriate. Or, alternatively, a larger-scale, extracellular view of the mechanisms in play in collagen formation and recruitment of the multifunctional stroma might be of interest. In particular, metabolic modelling has recently seen a number of advances: the construction of a whole human body metabolic model [50], and particular emphasis on metabolic interactions between cells at the multicellular level [52, 53]. We plan to keep exploring this diverse panel of methods in the future.

## Conclusions

We suggest that the Warburg effect—or ‘aerobic glycolysis’—abnormal lactate production from glucose—is correlated through specific elementary metabolic pathways to the formation of the tumor stroma, including collagen, which plays a key role in cancer progression and metastasis, inflammation markers and blood vessels. Metabolic pathways analysis suggests that the collagen production phenotype displayed by fibroblasts and Warburg effect might occur at the same time, and without extracellular import of the macromolecule’s main components, glycine and proline. During tumoral growth, amino acids might be recycled into cytokines to recruit immune cells and new blood vessels. Cancer cells are actors of the tumoral ecosystem: their wastes exert an ecological pressure on their environment and modify the surrounding landscape. The rerouting of resources and wastes by the newly formed stroma and vascular network has an impact on the larger scale of the organ and the full organism. Our tumor metabolic model encompasses the properties of the tumoral stroma at the cellular level, opening the road for further analyses of the impact of Warburg effect at the tissue level.

## Materials and methods

**Fig 8:**
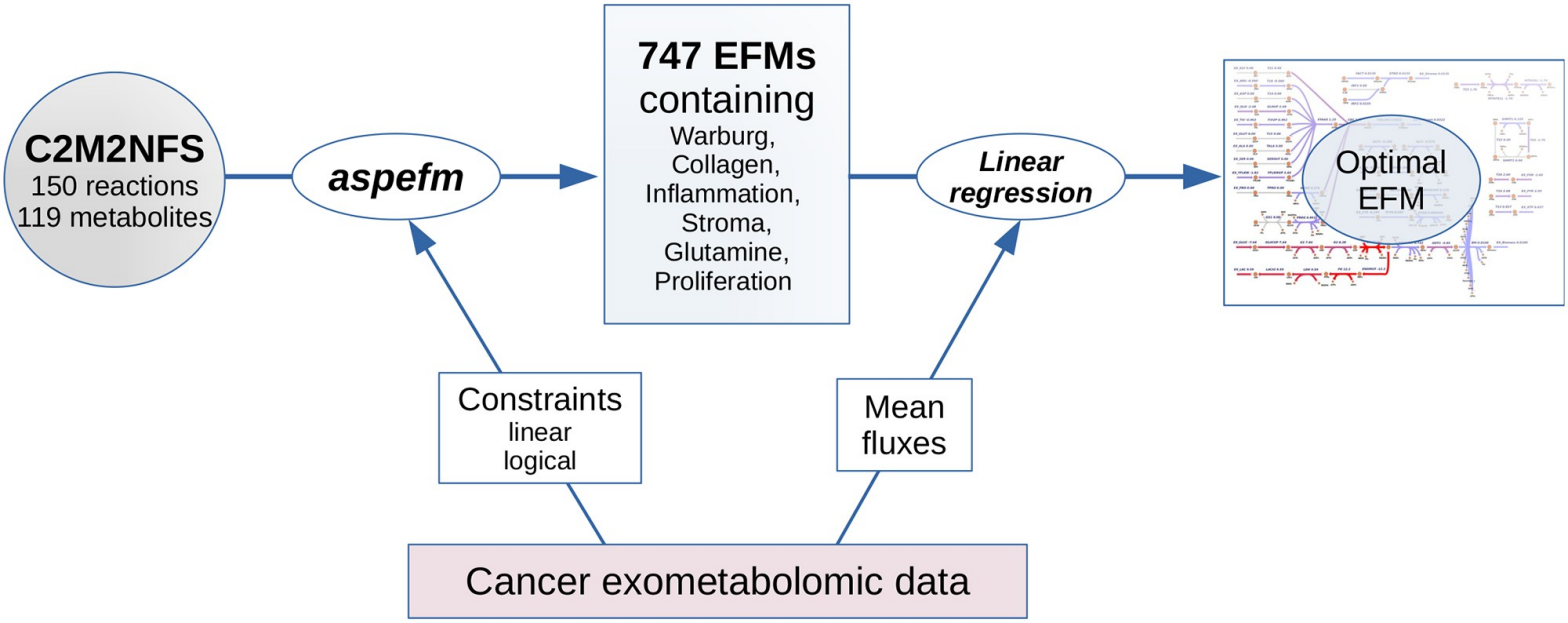
Description of the methods. Cancer exometabolomics data from the NCI-60 cell lines—from Jain and coauthors—is used to produce constraints for *aspefm* computation and for selecting the optimal EFM once the program executions were stopped.

We developed the C2M2NFS model as an extension of the C2M2NF model. The model comprises 150 reactions. In accordance with constraints describing Warburg effect, collagen production and inflammatory markers and growth factor VEGF-A synthesis, a subset of 747 EFMs was computed with *aspefm* [26]. Then, the best EFM was selected according to classic linear regressions of the solutions to the mean exometabolomics data from Jain and colleagues using Python package Scikit-Learn [54]. Finally, flux sampling and pFBA used for comparison to EFMs were performed with COBRAPy [20, 55]. A detailed look at our analysis workflow is presented in Fig 8.

Code, materials and methods for running the EFMs analysis and generating the figures are provided at https://gitlab.com/maxm41/tumoralstroma.

### EFMs computation with *aspefm*

Elementary Flux Modes are computed with *aspefm* [26], a hybrid logic programming tool based on *clingo*’s SAT solver technology [56] and leading linear programming tool *cplex* [57]. Logical and linear constraints can be added, and solutions obtained can be checked for elementarity using the stoichiometric matrix rank test [58]. Network reduction was performed using compression of reactions into ‘enzyme subsets’ [23, 58, 59].

To compensate for its lack of ability to complete enumeration, we ran several executions of *aspefm* in the time span of 3.5 days. All executions are non-deterministic due to the SAT-solver’s random decisions, therefore yielding us a non-exhaustive yet substantial sample of 747 EFMs respecting our constraints of interest for us to analyze.

*aspefm* can be found at https://github.com/maxm4/aspefm. Computations were performed using a server-hosted Intel^®^ Xeon^®^ E5–2609v2 2.5GHz processor.

### Logical constraints types and formulation

Logical constraints given in input to *aspefm* are detailed in Table 1. The constraints are separated into two types: hard constraints, which are forced inputs for the computation, and desired observations, not forcing any input but forbidding the opposite observation.

For example, the three hard constraints are: glucose must be consumed, lactate must be produced, glutamine must be consumed. And an example of expected observation would be aspartate should be consumed, *ie*. we forbid aspartate production.

Hard constraints are encoded as direct logical constraints: “forwards reaction must be active”, meaning “forwards reaction flux must be non null” (Eq 1) meanwhile desired observations are encoded by forbidding reactions from going in the backwards direction (Eq 2). We also provide the equivalent linear constraints for clarity.

$$\begin{array}{cccc} \forall r\in HardConstraints, & \nu_{r_{fwd}}>0\;↔z_{r_{fwd}} & \; & (r_{fwd},r_{bwd})\in Reversibles \end{array}$$ (1)

$$\begin{array}{cccc} \forall r\in DesiredObservations, & \nu_{r_{bwd}}=0\;↔\neg z_{r_{bwd}} & \; & (r_{fwd},r_{bwd})\in Reversibles \end{array}$$ (2)

Given that $\nu_{r}\;\forall r\in R$ represents the EFM value of each reaction *r* in the set of all reactions R, and that $z_{r}\;\forall r\in R$ are *aspefm* Boolean indicator variables of when a reaction is active, *ie*. when its EFM value is non null. Logical constraints are enforced by setting *aspefm* Boolean indicators to be *True* or *False*, taking advantage of the logic programming roots of the method.

For example, the hard constraint “lactate must be secreted” will be encoded by the constraint “flux going into forwards reaction of lactate production must be strictly positive”, while the expected observation “alanine must be secreted” will have the constraint “flux going into backwards reaction of alanine production must be null”.

### Linear constraints and size constraint

Adding linear constraints to the computation of EFMs such as operating costs is crucial for keeping EFM close to realistic conditions [21]. Namely, the constraints added are described by the following equations: Eq 3 constrains collagen production values to be over biomass production values; Eq 4 constrains inflammatory marker response to be over biomass production values. Concretely, Eqs 3 and 4 are akin to operating costs constraints; they impose that more metabolite units go into stroma response than into cell proliferation.

$$\begin{array}{cc} & \;\nu_{COLLAGEN}>\nu_{BIOMASS} \end{array}$$ (3)

$$\begin{array}{cc} & \;\nu_{STROMA}>\nu_{BIOMASS} \end{array}$$ (4)

Given that $\nu_{r}\;\forall r\in R$ represents EFM values for reactions *r* in the reactions set R.

In addition, a size constraint for number of reactions of EFMs was added:

$$\begin{array}{c} \;card\{r\in R\;|\;\nu_{r}\neq 0\}<60 \end{array}$$ (5)

The size constraint of equation Eq 5 constrains EFMs size to be of strictly below 60 active reactions. This bound was set in order to account for realistic enzyme usage, as enzyme utilization considerations help direct solutions towards better biological accuracy [17, 22]. Such a size constraint takes full advantage of the *aspefm* solver’s constraint programming origins.

### Incorporation of stroma formation in the metabolic model

The model C2M2NFS, for Central Carbon Metabolic Model with added Nitrogen, Folate, and Stroma formation, was expanded from the C2M2NF model [28] with: *(1)* collagen synthesis, *(2)* inflammatory response markers IL1*β* or TNF*α*, and *(3)* growth factor VEGF-A, linked to neoangiogenesis. The first condition *(1)* gets its own exchange reaction: EX_COLLAGEN, while the latter two *(2),(3)* are lumped together in the exchange reaction EX_STROMA.

Production of non-growth associated proteins is not usually taken into account into metabolic models, as these tend to only include purely metabolic processes. Protein production is occasionally integrated as a resource optimization procedure [60], meaning every enzyme catalyzing the metabolic processes must be synthesized. However, this requires a tremendous amount of experimental data to calibrate.

In the C2M2NFS model, protein production is done by simply redirecting amino acid metabolism to the production of proteins of interest using specific production reactions. Our proteins of interest are collagen and inflammation response markers, *ie*. IL1*β* or TNF*α*, and growth factor VEGF-A.

In order to define the C2M2NFS model, reactions were added to the C2M2NF model to incorporate missing amino acids and transporters: proline, histidine, alanine, asparagine, as well as associated reactions and pathways: alanine aminotransferase, asparaginase, histidine degradation pathway, etc. Pathways were retrieved from the Human map in KEGG PATHWAYS.

Collagen synthesis was defined from literature data as follows: the proportion of amino acids in collagen tripeptides was found to be roughly 33% Gly, 16% Pro+Hyp, and 50% rest [61]. Collagens come in various forms, including several types of chains of over a thousand amino acids [47].

We defined a polypeptide of collagen as 100 amino acid bricks. In order to model a reaction for collagen synthesis, we associated collagen formation to the amount of produced collagen polypeptides of 100 amino acids. This was done with the following reactions:

CBS: 0.33 Gly + 0.50 XYAA + 0.085 Pro + 0.085 Hyp → CBrick
COLLAG: 100 CBrick → Collagen

For other amino acids than Gly and Pro, except for Met, Cys, Asn, and His, which are four of the least common amino acids found in the X-Y part of collagen tripeptides [61], we assumed an equiprobability distribution. This corresponds to the XYAA metabolite, defined by the following reaction:

XYAAS: ALAc + ARGc + SERc + TIVc + YFLKWc + GLNc + ASPc +
GLUTc → 14 XYAA

Inflammatory markers IL1*β* and TNF*α*, and growth factor VEGF-A, were incorporated as protein synthesis reactions, based on the amino acid content of their consensus Uniprot FASTA protein sequence. Corresponding Uniprot entries were IL1B_HUMAN, TNFA_HUMAN and VEGFA_HUMAN.

Stoichiometric coefficients in the three following protein synthesis reactions correspond to amino acid proportions of the protein sequences. Thus, the stoichiometric coefficient next to the protein is the inverse of its length.

IL1B: 0.045 MET + 0.048 ALAc + 0.078 GLUTc + 0.13 TIV + 0.056 PROc
+ 0.275 YFLKW + 0.078 SERc + 0.048 GLYc + 0.045 ASNc + 0.074 ASPc
+ 0.067 GLNc + 0.019 CYSc + 0.022 ARGc + 0.015 HISc → 0.00371 IL1B
TNFA: 0.009 MET + 0.086 SERc + 0.163 TIV + 0.069 GLUTc + 0.06
ARGc + 0.03 ASPc + 0.245 YFLKW + 0.082 ALAc + 0.064 PROc + 0.073
GLYc + 0.056 GLNc + 0.017 CYSc + 0.017 HISc + 0.03 ASNc → 0.00429
TNFA
VEGFA: 0.034 METc + 0.03 ASNc + 0.237 YFLKWc + 0.065 SERc +
0.108 TIVc + 0.047 HISc + 0.034 ALAc + 0.056 GLNc + 0.065 PROc +
0.069 GLUTc + 0.06 GLYc + 0.034 ASPc + 0.082 ARGc + 0.078 CYSc →
0.00431 VEGFA

If one of the inflammation markers IL1*β* or TNF*α* is present in reasonable quantity through its production flux, and growth factor VEGF-A is also being produced, then an inflammatory response with neoangiogenesis is supposed on the model.

FACT: VEGFA → GrowthFactor
INF1: IL1B → Inflam
INF2: TNFA → Inflam
STROMA: Inflam + GrowthFactor→ Stroma

The resulting C2M2NFS model is of size 119 metabolites and 150 reactions, including 36 exchange reactions. After metabolic network compression, the network comprises 94 reactions, 66 internal metabolites, 25 external metabolites.

Finally, we tested the consistency of the stoichiometry of the models with tool GAMES, in an attempt to validate our new version of the model [62]. While both C2M2NFS and C2M2NF failed the linear programming stoichiometric consistency analysis, the GAMES algorithm itself did not report any error.

## Supporting information

S1 Fig Linear regression of the mean exometabolomics flux data to the parsimonious FBA solution. Represented reactions correspond to exchange reactions with non-null FBA values.
(TIF)

S2 Fig Parsimonious Flux Balance Analysis optimal solution obtained with the same constraints as for Elementary Flux Modes analysis. 70 reactions of most interest of C2M2NFS are shown, including most cytosolic transporters and some mitochondrial transporters but not mitochondrial TCA Cycle. Visualization of the reactions is done through the EscherPy Python package.
(TIF)

S1 File SBML file of the C2M2NFS model. Metabolic model in the community Systems Biology Markup Language format.
(XML)

S2 File 747 EFMs sampled on the C2M2NFS model. All EFMs computed respected constraints as indicated in Methods. EFMs correspond to rows and reactions to columns.
(CSV)

S1 Table Statistics of the main exchange fluxes for all 747 EFMs. Includes minimum, maximum, mean, median, standard deviation. Negative values indicates consumption, positive values indicates production.
(PDF)

S2 Table Scores of linear regression fit to the mean flux values of different types of cancer cell lines in the NCI-60 cancer cell lines data. Applied to the EFM with best regression fit to the global mean flux values of all 60 cell lines.
(PDF)
