# Supplementary figures and images for: Metabolic modelling links Warburg effect to collagen formation, angiogenesis and inflammation in the tumoral stroma
Source: PLoS One. 2024 Dec 3;19(12):e0313962. doi: 10.1371/journal.pone.0313962 (PMC11614220; doi:10.1371/journal.pone.0313962)

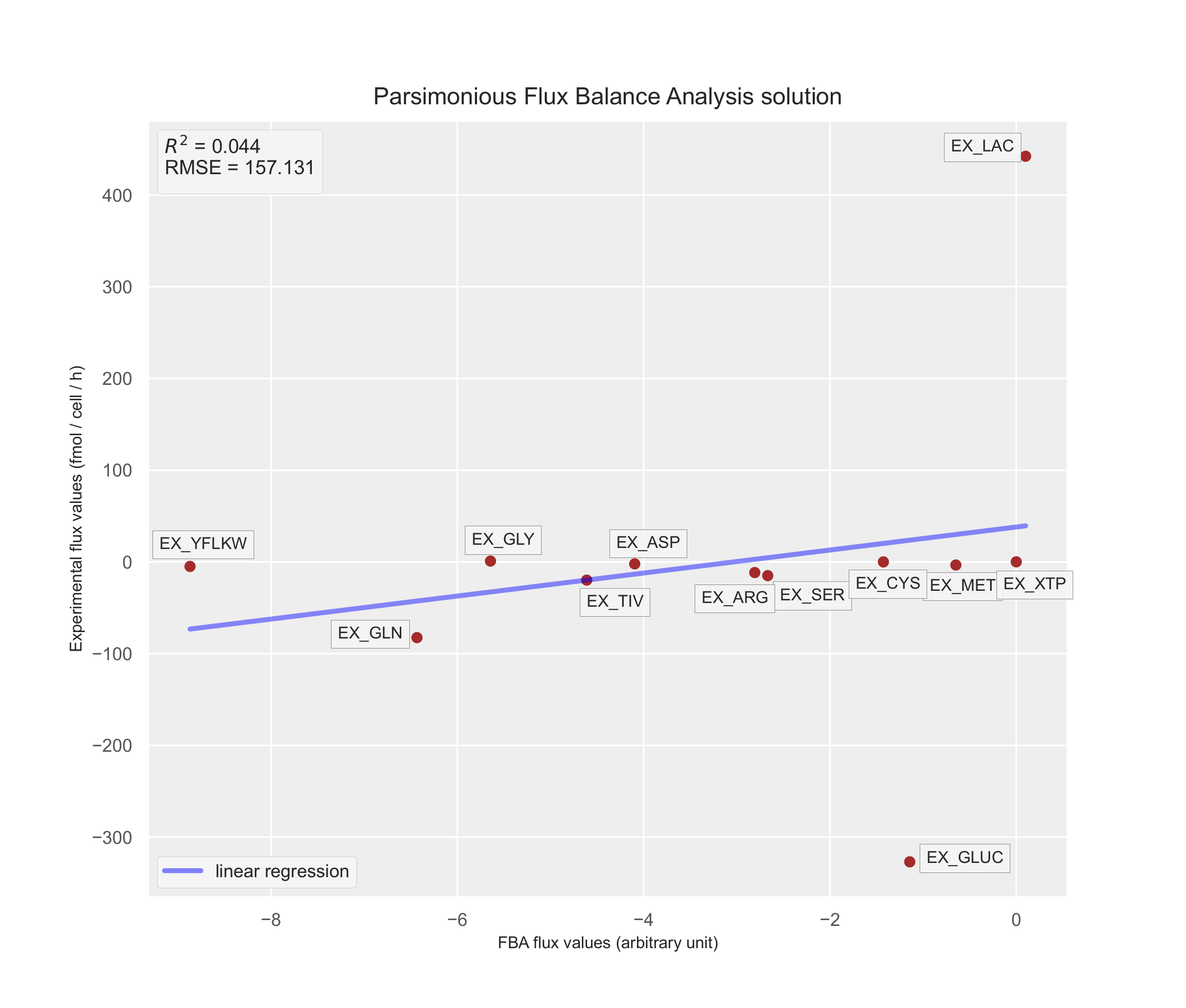

Supplement: S1 Fig — Represented reactions correspond to exchange reactions with non-null FBA values. (TIF) [file pone.0313962.s001.tif]

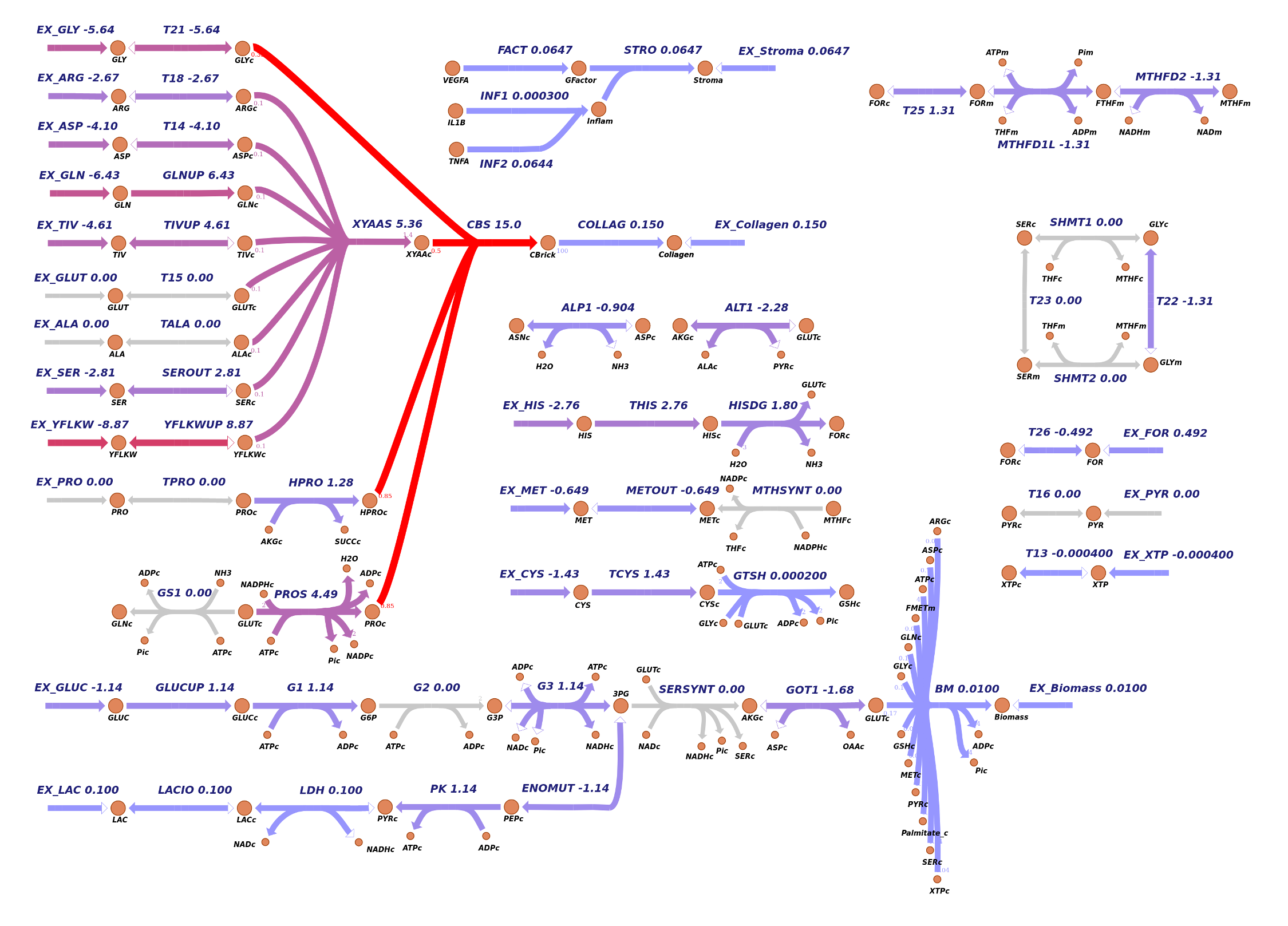

Supplement: S2 Fig — 70 reactions of most interest of C2M2NFS are shown, including most cytosolic transporters and some mitochondrial transporters but not mitochondrial TCA Cycle. Visualization of the reactions is done through the EscherPy Python package. (TIF) [file pone.0313962.s002.tif]
