# Supplementary material for: Metabolic modelling links Warburg effect to collagen formation, angiogenesis and inflammation in the tumoral stroma
Source: PLoS One. 2024 Dec 3;19(12):e0313962. doi: 10.1371/journal.pone.0313962 (PMC11614220; doi:10.1371/journal.pone.0313962)
Supplement: S1 Table — Includes minimum, maximum, mean, median, standard deviation. Negative values indicates consumption, positive values indicates production. (PDF) [file pone.0313962.s005.pdf]

	minimum	maximum	median	mean	standard deviation
**EX_GLY**	-11.7760	13.7858	0.0000	0.0361	3.3644
**EX_ARG**	-5.0745	0.0000	-0.8145	-1.0733	0.9929
**EX_ASP**	-12.8867	0.0000	-1.6067	-2.2061	1.9422
**EX_GLN**	-12.0169	-0.1697	-1.7679	-2.4997	2.2157
**EX_TIV**	-8.7957	-0.7183	-1.6904	-2.2185	1.6127
**EX_GLUT**	0.0000	6.2446	0.0000	0.1707	0.6644
**EX_ALA**	0.0000	4.9655	0.0000	0.0640	0.4226
**EX_SER**	-14.7054	0.0000	-1.5427	-2.5081	3.2967
**EX_YFLKW**	-14.8778	-1.3146	-3.0614	-4.0092	2.7661
**EX_PRO**	-8.5352	0.8575	-1.2870	-1.6464	1.5906
**EX_HIS**	-7.7354	-0.1635	-0.5035	-1.5421	1.8491
**EX_MET**	-1.2754	4.8228	-0.2290	-0.2083	0.6016
**EX_CYS**	-2.8155	-0.2429	-0.5519	-0.7241	0.5206
**EX_FOR**	-8.9515	12.0801	0.0000	0.4891	2.4040
**EX_PYR**	0.0000	4.8270	0.0000	0.1721	0.6349
**EX_XTP**	-0.0035	1.4832	0.3022	0.2963	0.2810
**EX_GLUC**	-8.5815	-0.5000	-2.3908	-2.8593	1.8851
**EX_LAC**	0.0091	14.9990	3.9917	4.7118	3.4683
**EX_Biomass**	0.0100	0.0879	0.0100	0.0185	0.0144
**EX_Stroma**	0.0110	0.1276	0.0248	0.0323	0.0236
**EX_Collagen**	0.0110	0.3125	0.0367	0.0583	0.0575
