# Supplementary material for: Metabolic modelling links Warburg effect to collagen formation, angiogenesis and inflammation in the tumoral stroma
Source: PLoS One. 2024 Dec 3;19(12):e0313962. doi: 10.1371/journal.pone.0313962 (PMC11614220; doi:10.1371/journal.pone.0313962)
Supplement: S2 Table — Applied to the EFM with best regression fit to the global mean flux values of all 60 cell lines. (PDF) [file pone.0313962.s006.pdf]

| | Number of cell lines | Linear regression RMSE | Linear regression $R^2$ |
|---|---|---|---|
| **All cell lines** | 60 | 26.9102 | 0.9813 |
| **Colon** | 7 | 28.7879 | 0.9812 |
| **Leukemia** | 6 | 19.3316 | 0.9745 |
| **Lung** | 9 | 40.9490 | 0.9797 |
| **Prostate** | 2 | 33.1043 | 0.9789 |
| **Ovarian** | 7 | 25.9862 | 0.9818 |
| **Breast** | 6 | 31.3463 | 0.9803 |
| **Melanoma** | 9 | 23.2846 | 0.9818 |
| **Central Nervous System** | 6 | 19.9142 | 0.9789 |
| **Renal** | 8 | 28.8023 | 0.9770 |
